# Leveraging Linked Ontario’s Health-administrative Data to Evaluate Internet-delivered Cognitive Behavioural Therapy (iCBT) in routine care

**DOI:** 10.23889/ijpds.v9i5.2523

**Published:** 2024-09-18

**Authors:** Evgenia (Jenny) Gatov, David Wiljer, Gillian Strudwick, Onil Bhattacharyya, Paul Kurdyak

**Affiliations:** 1 ICES; 2 Institute of Health Policy, Management and Evaluation; 3 University Health Network; 4 Centre for Addiction and Mental Health; 5 Women's College Hospital

## Objective

Virtual interventions show promise for meeting growing mental health (MH) service demands. In 2020, Ontario launched Internet-delivered Cognitive Behavioural Therapy (iCBT), an evidence-based, self-led, asynchronous intervention for anxiety and depression. While its efficacy has been demonstrated in randomized controlled trials, we examine its real-world reach and treatment completion in routine care.

## Approach

Linking iCBT records with health-administrative databases, we examined the characteristics (clinical, sociodemographic, health service use) of individuals receiving iCBT (via a pilot program), compared to fully-synchronous CBT (standard of care), ascertained who is more likely to receive either modality, and determined factors associated with treatment completion using Logistic regressions adjusted for sociodemographics, baseline depression (PHQ-9) and anxiety (GAD-7), and outpatient and acute MH-related service use one year prior to enrollment.

## Results

Among N=167 individuals receiving iCBT at the Centre for Addiction and Mental Health (Jan 2020-Aug 2021) and N=300 controls receiving fully-synchronous CBT via Ontario’s Structured Psychotherapy Program, we found that older individuals, those with lower baseline anxiety, and those with greater prior MH service use were more likely to receive iCBT. Intervention modality was the only significant predictor for treatment completion, with 51% lesser odds among iCBT clients (ORadjusted=0.49, 95%CI 0.31-0.76).

## Conclusions

While certain demographic characteristics differentiate iCBT recipients from those receiving standard CBT, treatment adherence is lower in self-led, asynchronous therapy.

## Implications

This study examines iCBT in routine care and informs its wider implementation so it can reach the target population, ease access burdens, and improve MH service delivery. Future work will examine treatment effectiveness.

